# Advances in the application of 3D tumor models in precision oncology and drug screening

**DOI:** 10.3389/fbioe.2022.1021966

**Published:** 2022-09-28

**Authors:** Xiaoyong Guan, Shigao Huang

**Affiliations:** ^1^ Department of Clinical Laboratory, The First Affiliated Hospital of Guangxi University of Science and Technology, Liuzhou, Guangxi, China; ^2^ Department of Radiation Oncology, The First Affiliated Hospital, Air Force Medical University, Xi’an, China

**Keywords:** 3D tumor cell culture, 3D tumor sectioning, antitumor drugs screening, tumor organoids, precision oncology

## Abstract

Traditional tumor models cannot perfectly simulate the real state of tumors *in vivo*, resulting in the termination of many clinical trials. 3D tumor models’ technology provides new *in vitro* models that bridge the gap between *in vitro* and *in vivo* findings, and organoids maintain the properties of the original tissue over a long period of culture, which enables extensive research in this area. In addition, they can be used as a substitute for animal and *in vitro* models, and organoids can be established from patients’ normal and malignant tissues, with unique advantages in clinical drug development and in guiding individualized therapies. 3D tumor models also provide a promising platform for high-throughput research, drug and toxicity testing, disease modeling, and regenerative medicine. This report summarizes the 3D tumor model, including evidence regarding the 3D tumor cell culture model, 3D tumor slice model, and organoid culture model. In addition, it provides evidence regarding the application of 3D tumor organoid models in precision oncology and drug screening. The aim of this report is to elucidate the value of 3D tumor models in cancer research and provide a preclinical reference for the precise treatment of cancer patients.

## Introduction

**FIGURE 1:**
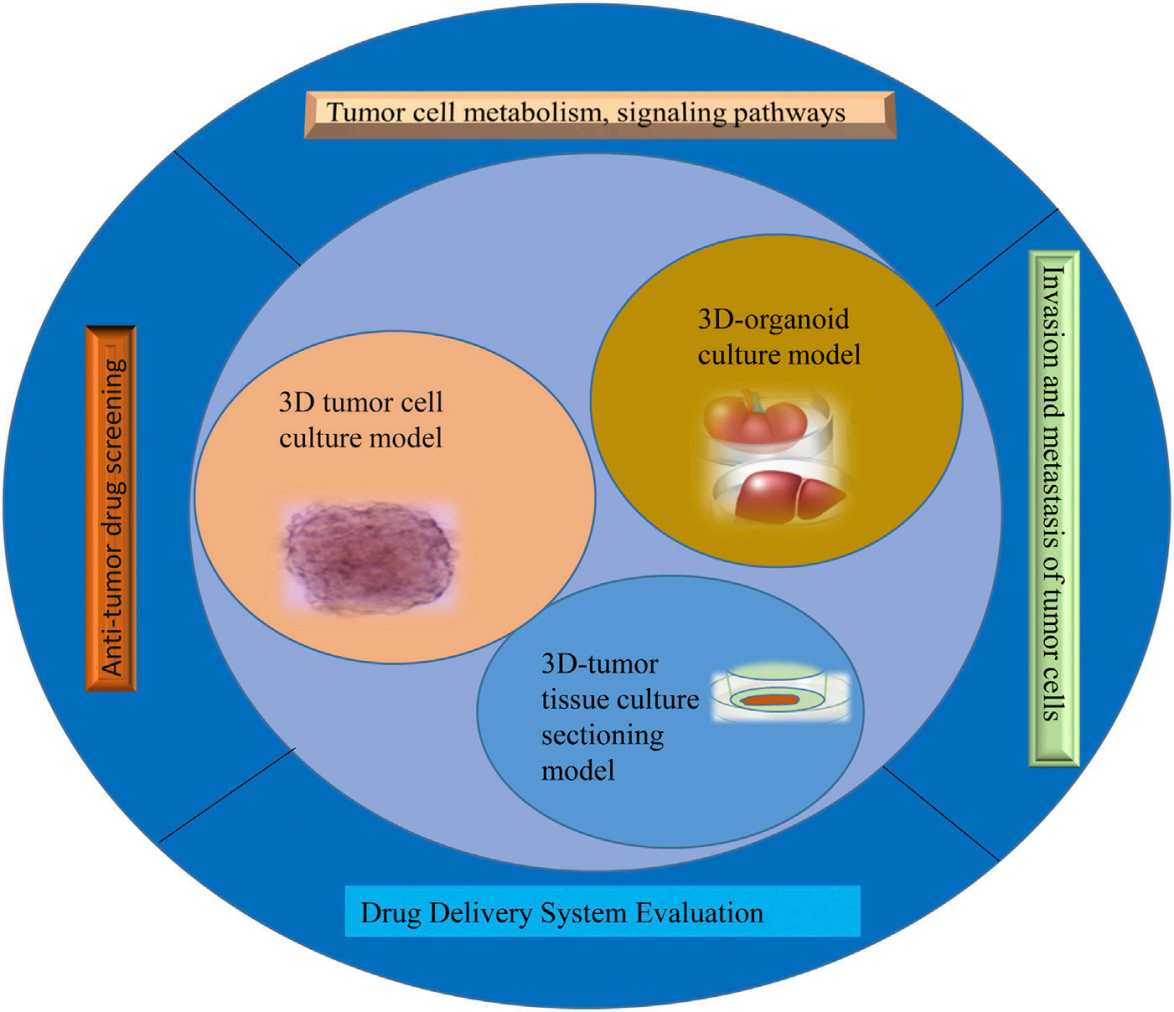
Schematic of 3D tumor models in precision oncology and drug screening.

The success rate of the clinical development of antineoplastic drugs is much lower than that of other drugs. The reason is that tumors are a far more complex disease than is retained, and their occurrence, growth, and metastasis are related not only to tumor cells but also to their environment (Swann and Smyth, 2007). The tumor microenvironment is the ecological environment on which tumor cells depend for survival and development (Anderson and Simon, 2020). In this environment, tumor cells come into contact with each other and with immune cells (Sharonov et al., 2020), tumor-associated fibroblasts (Truffi et al., 2020), endothelial cells (Sobierajska et al., 2020), inflammatory cells (Turley et al., 2015), and noncellular components, which can significantly affect the biological properties of tumor cells, such as their polarity, structure, resistance, migration, and invasion (Turley et al., 2015). The traditional tumor monolayer cell culture model uses the nature of cell adherent growth to form a dense tumor monolayer cell structure; these culture methods are simple to operate with and low cost, but 2D cell culture does not reflect the *in vivo* environment in terms of morphology, structure, and function and cannot simulate the three-dimensional characteristics of tumor heterogeneity and the microenvironment (Tuveson and Clevers, 2019). Therefore, the single-layer planar culture model is insufficient for predicting the real condition of drugs in tumor tissue, and the experimental results are rarely consistent with the results of clinical trials, resulting in a low success rate in antitumor drug development (Xu et al., 2018a). This study summarizes the 3D tumor cell culture model, 3D tumor slice model, and organoid culture model and reviews their application in antitumor drug research (Figure 1). It aims at elucidating the value of 3D tumor models in cancer research and provides a preclinical reference for the precise treatment of cancer patients.

**FIGURE 2:**
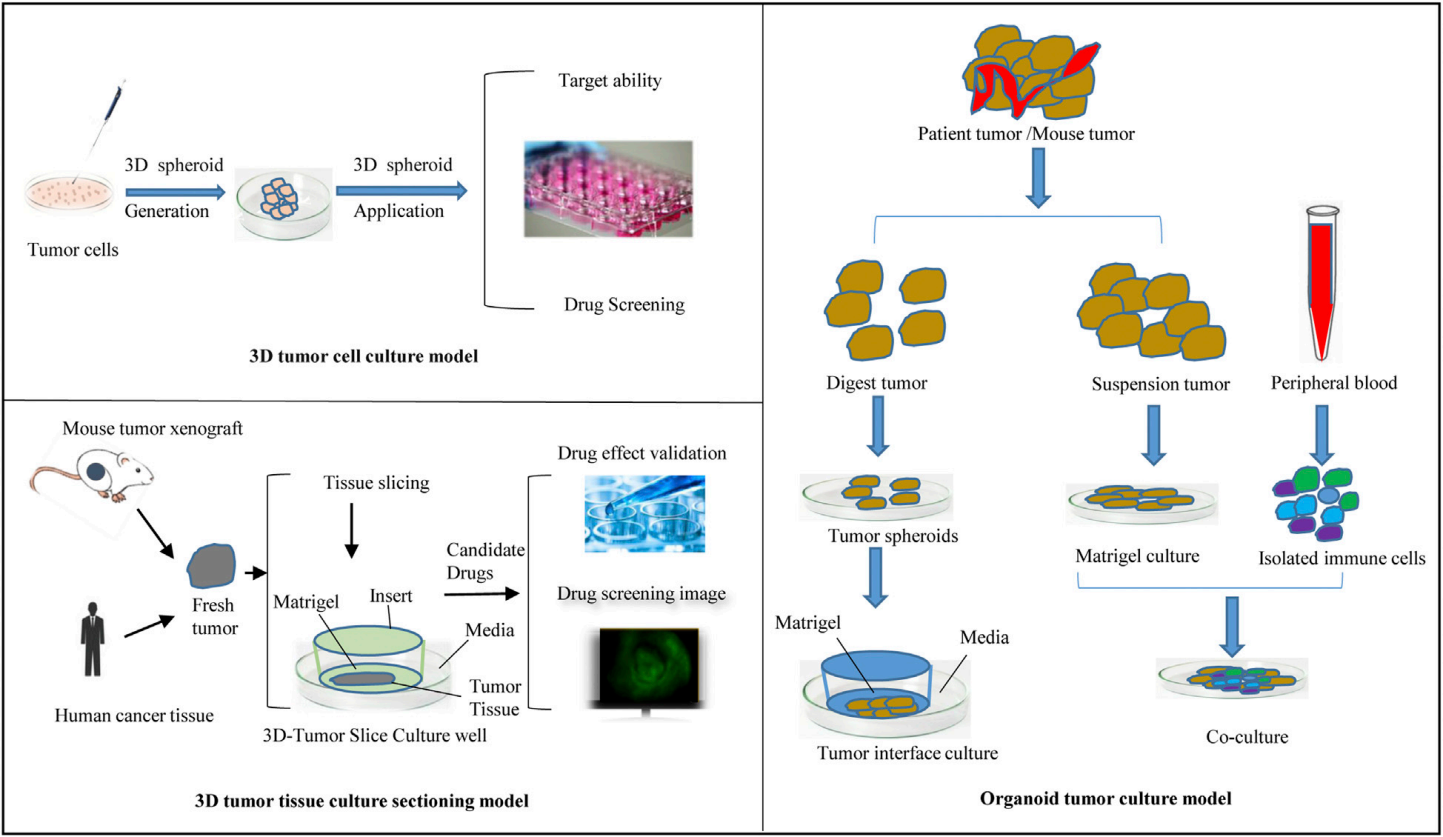
Types of 3D tumor culture process in xenograft and human model.

Culture to promote our understanding of complex biological processes, and address the limitations of many traditional 2D cell cultures. At present, organoid models derived from 3D cell culture are gradually being used in a variety of research applications, including cell biology, regeneration methods, precision medicine, and drug toxicity and efficacy testing, showing great application potential. The use of 3D cell culture as the main cell culture process in the future will undoubtedly become a major trend, but there are still some challenges for scientists to solve before this technology can be widely used (Neal et al., 2018). As cell culture media components, such as scaffolds and gels, continue to evolve, 3D tumor cell models can be used to simulate three-dimensional spaces and microenvironments similar to tumors *in vivo*. This technology has gradually become the most promising cell research model and is widely used in antitumor drug research. This section provides a brief introduction to commonly used 3D tumor cell models and their applications in antitumor drug research (Figure 2).

### Multicellular tumor spheroid culture model

**FIGURE 3:**
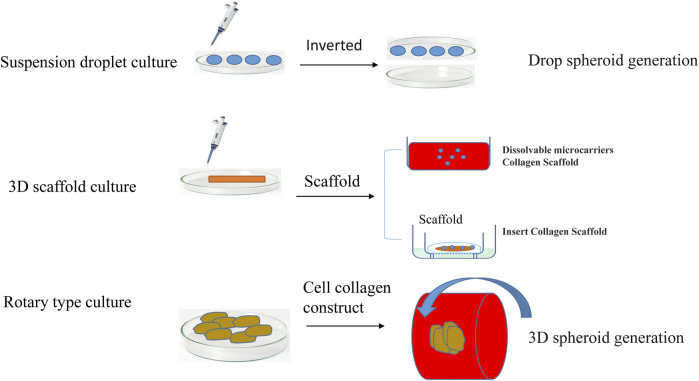
The typical spheroid formation methods and materials-based spheroid formation systems.

At present, the cell model that can be used to best characterize tumor organs is multicellular tumor spheroids (MCTSs). MCTSs are classified according to the culture method, mainly including suspension culture, rotary culture, and scaffold culture (Figure 3). Suspension culture occurs when a dish rich in cell droplets is flipped, and surface tension and gravity promote the formation of a suspension of cells that aggregate into tumor spheres (Tung et al., 2011). Suspension droplet culture does not require special equipment, the culture cost is low, and the size of the tumor ball is relatively uniform, but the traditional suspension drop method is not suitable for long-term culture due to evaporation; additionally, it is difficult to keep the original microenvironment features, the tumor spheroids need to metastasize after formation and then cultured, and the isolation and purification procedures are more complicated. Tumor cell aggregates are later cultured using 96-well or 384-well plates to more accurately control the size of the tumor spheres. Suspension culture promotes the spontaneous aggregation of tumor cells by reducing the effects of gravity during the formation of multi tumor cell spheroids (Lee et al., 2007). Suspension cultures are easy to perform and no special equipment is required, but the culture cycle is long, and the tumor bulb size is not easy to control.

By maintaining a certain stirring rate and promoting the transport of nutrients and metabolic waste, the interaction of the matrix is maintained. This method can be used to culture a wide variety of cell types but is complex and expensive until making it particularly unsuitable for shear-sensitive or low-adhesion cells. Multiple cell co-culture models based on scaffolds have been widely studied. Scaffolder-based 3D models are embedded outside the cells of the simulated tumor with cells or clusters of cells. Scaffold culture promotes the adherence of tumor cells to the scaffold fiber culture through the continuous division of cells; the cells fill the gaps in the scaffold and form a cell ball. The technique is simple, can be used to culture a variety of cell types, and can also utilize growth factors, cytokines and other features of the tumor cell microenvironment (Tsai et al., 2022). The most commonly used scaffold is collagen. The disadvantage of stent culture is that the stent is relatively expensive, and the scaffold component has a greater impact on cell culture. Stent culture is the most commonly used and well-studied method of tumor spheroid culture.

Since single tumor spheroid formation cannot reflect the tumor microenvironment (Pang et al., 2019), a hybrid system with different cells were applied in anti-tumor drug evaluation to overcome the limitation of tumor, and the effect on tumor cell properties was also studied. Generally, a hybrid co-culture system from different cells is difficult to establish. However, to some extent, the hybrid co-culture system reflects the interaction between tumor and its surrounding microenvironment *in vitro*. It can be used to screen drugs. Kim et al. (2012) developed cellular complexes containing human hepatocellular carcinoma cell line (Hep-G2) and rat insulin-secreting cell line (RIN-5F) by using a co-culture hybrid cellular spheroids model (HCSs). They found that the insulin and albumin levels in the HCSs were considerably higher than those in single tumor spheroid formation. Thus, a hybrid system had its advantage in immune effect in the whole tumor microenvironment.

### Tumor-on-a-chip culture model

With the rapid development of chip-making technology, production materials, and detection methods in microelectronic processing technology, generating a culture model of cells and even “organs” on chips for screening drugs is of great importance (Albanese et al., 2013). The tumor on a chip fabrication and design are mainly constituted of a cell culture/tissue chamber and a channel for delivering the medium. Through adjusting microchannels on the chip to simulate the structure and state of solid tumor tissue, which can be used to evaluate and screening drugs. Aung et al. (2020) has implanted breast cancer cells in multilayer paper chips for perfusion culture, simulated the structure of 3D solid tumors, established a barrier for the exchange of materials between tumor cells and the external environment, simulated capillaries *in vivo* with microchannels in the chip, adjusted the perfusion speed of microchannels on the chip, and simulated the relatively insufficient state of vascular oxygenation in solid tumor tissue. After the perfusion culture was completed, the tumor tissue is decomposed by splitting the multilayer membrane and detecting the tumor metabolic state at different depths. Experiments have proven that this multilayer paper chip promotes fast external growth and slow internal growth in solid tumors, and internal hypoxia necrosis occurs in the presence of tumor microenvironments with low oxygen and low pH (Kang et al., 2016). Microfluidic cell culture (Huh et al., 2011; Mehling and Tay, 2014; Ng et al., 2015; Bale and Borenstein, 2018) on a chip is a technique for cell culture on a chip with a cell culture chamber and a channel for delivering media (Walsh et al., 2009). Microfluidics can be used to precisely control the perfusion speed of the injected and transported medium between cells and extracellular matrix simulation materials. Unlike traditional cell experiments, microfluidic chip culture can be used to reflect the interaction between cells, the cell microenvironment, and the concentration gradient formed by various cytokines, etc., with strong controllability, large data volume, and reliable results (Huh et al., 2011; Polidoro et al., 2021). Recently, using a 3D microfluidic system in the presence of fibronectin to explore the crosstalk between fibroblasts and breast cancer cells (MDA-MB-231), the results showed that the capability of the model to pinpoint the contribution of different components of the tumor microenvironment (TME) (Lugo-Cintrón et al., 2020). However, shortcomings, such as difficulties in chip production and application, have limited the promotion of this model.

### The hydrogel-based 3D bioprinting tumor models

3D bioprinting is a 3D printing technology that uses bio-ink loaded with cells as a printing material to produce biologically active tissue and organ scaffolds and chips (Mandrycky et al., 2016; Gungor-Ozkerim et al., 2018; Heinrich et al., 2019; Murphy et al., 2020). At present, 3D bioprinting combined with cells has gradually become a research hotspot, which has a good expected application and prospect in tissue engineering regeneration, drug screening, disease treatment and other aspects (Annabi et al., 2014). Hydrogel materials contain a large amount of water, which requires mild conditions for printing. There are three main working principles applied to hydrogel, including inkjet bioprinting system based on materials and adhesives, extrusion nozzle bioprinting system and bioprinting system based on photopolymerization. The printing methods based on photopolymerization included stereo lithography, two-photon polymerization and laser-induced transfer (Murphy et al., 2020). 3D printing technologies with various working principles have been widely used in hydrogels. In addition, 3D bioprinting combined with microfluidic technology can create complex flow channels/chambers and functional biological structures with 3D heterostructures, cell placement, and tissue specificity to more closely resemble real tissues or organs (Heinrich et al., 2019). Colosi et al. (2016) used the mixture of alginate and GelMA to develop a bioink with low viscosity, combined with a microfluidic platform to form a microfluidic system with accurate and controlled deposition. It could promote the propagation and migration of cells inside biological structures. Based on this, they created a non-uniform 3D tissue model *in vitro* to simulate native tissues. It can be used in drug development.

### 3D tumor tissue culture sectioning model

The tumor tissue culture model is an aged tumor three-dimensional model in which a block of tumor tissue is placed on a dedicated porous culture plate, an appropriate medium is added, and the tumor tissue is cultured before it can be used for experimental studies (Huh et al., 2011; Polidoro et al., 2021) (Figure 2). Unlike traditional monolayer cell culture, this method can be used to accurately predict the sensitivity of tumor tissue to antitumor drugs while maintaining the original tumor structure and is used to screen antitumor drugs and guide personalized administration (Ravi et al., 2015; Zuppinger, 2019; De León et al., 2020; Jensen and Teng, 2020; Habanjar et al., 2021). The disadvantages of this model are the lack of reproducibility due to the natural heterogeneity of donor tissues and the difficulty of applying the required techniques, such as imaging and flow cytometry; thus, the application has limitations.

Three-dimensional tumor slide culture (3D-TSC) can be used to quickly and accurately reproduce the high complexity of tumors *in vivo* for drug screening, especially for immunotherapy drugs (Sivakumar et al., 2019; Nishida-Aoki et al., 2020). 3D-TSCs are produced by cutting slices of a fresh tumor without prior treatment while preserving the tumor structure, stroma, and TME. In our previous study (Huang and Zhao, 2020; Xing et al., 2021; Huang and Zhao, 2022; Peng et al., 2022), tumor slices from colon cancer and liver cancer patients generated after surgery were used, and nanomedicine combined with immunotherapy was applied to this platform to test toxicity and efficiency. Other results also showed that the components of the TME, including T cells and macrophages, could survive in the 3D-TSC platform for more than a week after 3D-TSC culture, thus allowing the study of the immune environment (Kenerson et al., 2021). The early drug screening results of 3D-TSC showed similar results to those of PDOs, and the generation of 3D-TSC can produce faster results, providing rapid and accurate guidance strategies for clinical patients during treatment decision-making, especially regarding the response of the tumor to various new immunotherapies (Ravi et al., 2015; Habanjar et al., 2021). Figure 2 summarizes the flow of the use of 3D-TSCs in a preclinical tumor model.

### Organoid culture model

The development of organoid technology has laid the foundation for the cultivation of tumor organoids. Tumor organoids are mainly generated either by gene editing normal tissue-derived organoids or by culturing directly from tumor tissue (Lee et al., 2007; Li et al., 2018; Jensen and Teng, 2020). Tumor tissue extraction is less restrictive, and the procedure inolves surgery, puncture biopsy, circulating tumor cells, pleural effusion, and cell brushing. The method of establishing organoids from tumor tissue is summarized as follows: after obtaining surgical excision of tissue, first, fat and muscle tissue are removed from cancer tissue, followed by trypsin (and) or collagenase treatment to digest the tissue according to the characteristics of the tissue. The cell suspension is resuspended with stromal colloid after passing through the cell screen and finally injected into the culture plate, and the appropriate medium is added for subsequent culture. The composition of the medium varies according to the characteristics of different cancer species, which are usually based on several types of factors, including V82 signaling pathway activators, tyrosine receptor kinase ligands, and signaling pathway inhibitors (Neal et al., 2018; Nuciforo et al., 2018; Cattaneo et al., 2020; Lu et al., 2021). During organoid research, clonal drift can be avoided by passing the entire Petri dish, using earlier generations of organoids, and reducing the number of passages.

Tumor research requires identifying the model that is closest to the real state of the tumor *in vivo* as the object of study. The traditional tumor research models mainly include cell culture, transgenic mice, and human tumor xenotransplantation models, each with advantages and disadvantages (Neal et al., 2018; Cattaneo et al., 2020; Yuki et al., 2020; Zhang et al., 2022a). Cell culture cycles are short and inexpensive, but gene drift occurs after multiple passages, resulting in changes in cell phenotype and drug sensitivity. In addition, cell culture cannot be used to simulate the interaction between cancer cells and the microenvironment, and it is difficult to replicate the patient’s treatment response. A human tumor can be directly transplanted into an animal for modeling, which preserves the heterogeneity of the tumor, but exhibits the limitations of a long modeling cycle and high cost (van de Wetering et al., 2015; Yan et al., 2018; Tuveson and Clevers, 2019; Yoshida, 2020; Yuki et al., 2020; Xu et al., 2021). In addition, the inability to maintain the matrix composition of human tumors may lead to mouse-like evolution of the tumor tissue. Finally, due to species differences, transgenic mice cannot be used to fully reflect the genetic and proteomic complexity of human tumors, and the results of these experiments lack consistency with human disease progression and drug sensitivity, which weakens the application value of transgenic mouse models to a certain extent.

Organoids have the combined advantages of the above culture methods and have unique advantages in tumor research (Neal et al., 2018). First, the core advantage of tumor organoid research is that it preserves the heterogeneity of the original tumor. Second, tumor organoids enable the expansion of small tumor samples from different sources (e.g., from puncture biopsy, pleural effusion, circulating tumor cells), which can be used to model different stages of tumors. Unlike tumor cells cultured using traditional (Zhang et al., 2022a) methods, tumor organoids exhibit genomic and transcriptome stability, which result in the maintenance of the protein expression pattern of the original tissue. Tumor cell organoids are implanted in mice after culture, and the modeling speed and success rate are high. It is worth noting that organoid technology can be used to separately model cancerous tissue and normal tissue obtained from the same patient source, providing a reliable control during tumor research.

## Application of 3D tumor organoids models

### Antitumor drug screening

The tumor microenvironment significantly affects targeted drug therapy, and cell growth performed in traditional 2D culture models does not exhibit three-dimensional spatial structure, resulting in antitumor drugs with false-positive results entering clinical trials, with a high failure rate and a large time and energy costs. Part of the reason is that the early trials using monolayer planar cells as the subject of the study were poorly designed, and the screening efficiency of antitumor drugs was low. Ingeson-Carlsson et al. (2015) compared the effects of RAF and MEK inhibitors to BRAF inhibitors on thyroid cancer drug responses during experiments on tumor cell migration in 2D and 3D cultures. They have shown that RAF and MEK inhibitors block the invasion of thyroid cancer spheroids (SW1736) but have no effect on the migration of SW1736 monolayer cells. Other studies (Godugu et al., 2013; Godugu and Singh, 2016) showed that an *in vitro* 3D model of antineoplastic drug screening was developed with the AlgiMatrix™ scaffold, in which cytotoxicity can be determined by a cell proliferation test and the effectiveness of antineoplastic drugs can be evaluated based on the spheroid number and size distribution. By immunohistochemistry and RT‒PCR evaluation, the assessment of anti-apoptotic markers and the comparison of 3D model and 2D monolayer model results, the data showed that 3D *in vitro* trials of cultured antineoplastic drugs were more suitable for the screening and evaluation of antineoplastic drugs. Active tumor-stromal interactions in hepatocellular carcinoma showed weaker efficacy than in two-dimensional monolayer cultured cell and different potency in 3D spheroid models, demonstrating the great potential of 3D multicellular spheroid models in the discovery and development of anticancer drugs (Vinci et al., 2015; Nath and Devi, 2016; Rodríguez-Dorantes et al., 2021; Zaki et al., 2021). The antitumor drug screening application are summarized in Table 1 **.**

**TABLE 1:** Various applications of 3D tumor organoid models in drug screening and mechanism.

Year	Finding	Method	Cancer type	Reference
2015	RAF and MEK inhibitors block the invasion of thyroid cancer spheroids (SW1736) but have no effect on the migration of SW1736 monolayer cells	2D and 3D spheroids cultures	Thyroid cancer	Ingeson-Carlsson et al. (2015)
2016	The effect of EphA2 receptor targeted docetaxel-loaded nanoparticles on MDA-MB-468 TNBC cell lines	Algimatrix™-based 3D Cell culture system	Non-small-cell lung cancer (NSCLC) models	Godugu and Singh (2016)
2021	A protocol to using prostate cancer cell lines (lncap, PC3, vcap) to improve research considering tumoral heterogeneity role	3D model of spheroids	Prostate cancer	Rodríguez-Dorantes et al. (2021)
2021	Corroborated using Hep3B homotypic spheroids cultured in LX2 (human hepatic stellate cell line) conditioned medium (CM). LX2 CM triggered the proliferation of Hep3B spheroids compared to control tumor spheroids	3D homotypic and heterotypic tumor spheroids by immobilizing cell suspensions on the lids of standard 10 cm^3^ Petri dishes	Hepatocellular carcinoma	Zaki et al. (2021)
2021	Deconvolute bulk data from endometrial cancers and endometriotic lesions, illuminating the cell types dominating in each of these disorders	Generated dense single-cell and spatial reference maps of the human uterus and 3D endometrial organoid cultures	Benchmark of the endometrial organoids	Garcia-Alonso et al. (2021)
2017	Narrow matrix-enclosing model, malignant tumor cells reencode specific malignancy genes, generate a structure that mimics blood vessels, and promote the spread of cancer cells through the blood to other areas of the body	Customized 3D collagen matrix	Solid human cancers	Velez et al. (2017)
2014	iRGD-PPCD antitumor drug delivery system exhibits higher tumor permeability comparing to RGD-PPCD in 3D spheroids but has no difference in 2D cell model	3D spheroids	C6 glioma tumor	Wang et al. (2014)
		2D cell model		
2016	Specific methods and recommend the use of adapted and standardized spheroid generation protocols for each cell line.	Different spheroid generation models including hanging drop, liquid overlay and suspension culture	Breast cancer tumor	Froehlich et al. (2016)

Abbreviation: MEK, mitogen-activated protein kinase; iRGD, internalizing RGD; PPCD, PEG-PAMAM-cis-aconityl-DOX

### Tumor cell metabolism and signaling pathways

Tumor signaling pathways and interventions do occur in monolayer planar cell culture (Rodenhizer et al., 2016; Flint et al., 2020; Garcia-Alonso et al., 2021; Yi et al., 2021); (Table 1)**.** However, studies have shown that signaling pathway activation in 3D cultured multicellular tumor spheres is significantly different from that in monolayer 2D planar cells due to the death receptor DR4. One of the reasons for the difference in signaling pathways between 2D and 3D cultured cell models is the different tumor microenvironment. Extracellular matrix components, such as adhesin and fibronectin, provide key signals that affect cellular function by activating intracellular signaling pathways, and integrins located at the cell-matrix interface are also activated by changes in extracellular matrix composition (Rashidian and Luo, 2016; Rodenhizer et al., 2016; Yi et al., 2021). Hsu and Huang (2013) developed a dynamic 3D multicellular spheroid (MSCs) using a unique biomaterial, and its differentiation ability was observed to be transmitted with Wnt signaling. This finding is not observable in conventional monolayer culture cells; suitable 3D cell spheres can be used to detect the role of Wnt signal regulation in different extracellular environments and can be used to study the behavior of tumor stem cells. The function and properties of 3D tumor spheres are more similar to those of solid tumor tissue than monolayer planar cell cultures.

### Invasion and metastasis of tumor cells

Invasion and metastasis of tumor cells, including the interaction of tumor cancer cells with the *in-situ* cell microenvironment and metastatic microenvironment, is an extremely complex process (Horie et al., 2012; Horie et al., 2015; Dwyer et al., 2016; Cattin et al., 2018; Du et al., 2018; Ilina et al., 2020; Colombo and Cattaneo, 2021). The main reason for the lack of recent research resides in the absence of an ideal model to simulate this complex physiological environment (Salgueiredo-Giudice et al., 2012; Schreiber-Brynzak et al., 2015; Rodenhizer et al., 2018). Velez et al. (2017) used a customized 3D collagen matrix to study the metastasis mechanisms of malignant cell tumors. They have shown that in a relatively narrow matrix-enclosing model, malignant tumor cells re-encode specific malignancy genes, generate a structure that mimics blood vessels, and promote the spread of cancer cells through the blood to other areas of the body, and this property of tumor cells has never been demonstrated in traditional monolayer cell culture methods; thus, the key to successful tumor cell experiments is to establish a 3D cell culture model that more accurately simulates the *in vivo* environment (De León et al., 2020; Garcia-Alonso et al., 2021). Metastasis of tumor cells model are summarized in Table 1.

### Drug delivery system evaluation

Using tumor-targeted nanocarriers, such as liposomes, nanoparticles, or micelles, produces unique advantages for the delivery of antitumor drugs or genes, such as increasing the *in vivo* circulation time, increasing tumor site accumulation, and reducing toxicity to normal organs (Campisi et al., 2012; Shin et al., 2013; Li et al., 2018; Carey-Ewend et al., 2020; Nii et al., 2020; Bartusik-Aebisher et al., 2021; Borodina et al., 2021; Bromma et al., 2021; Foglietta et al., 2021). RGD peptides are a kind of peptide that contain Arg-Gly-Asp sequence, internalizing RGD peptide (iRGD) can increase drug penetration into extravascular tumor tissue. PEGylated PAMAM dendrimer (G4) with DOX conjugated by acid-sensitive cis-aconityl linkage (PEG-PAMAM-*cis-aconityl*-DOX, PPCD) was modified by a RGD cyclopeptide. Wang et al. (2014) used C6 glioma 3D spheroids to show that the iRGD-PPCD (internalizing RGD peptide with PEG-PAMAM-cis-aconityl-DOX, PPCD antitumor drug delivery system is similar to RGD-PPCD, which exhibits higher tumor permeability, and when this group of experiments was performed in a 2D cell model, there was no significant difference in the results of analyses of *in vitro* cytotoxicity and cell uptake using the two delivery systems^.^ This finding shows that the 3D model has incomparable advantages in the evaluation of the permeability of antitumor drug delivery systems. Traditional 2D monolayer cell culture systems have many limitations, and 3D tumor cells can be used to mimic more complex cellular heterogeneity and interactions as well as tumor microenvironmental conditions (Wang et al., 2014; Xu et al., 2014; Wan et al., 2017; Tortorella et al., 2021). Although 3D models have incomparable advantages over 2D models, 3D cell culture models also have limitations that hinder their further application. First, not all tumor cells can be cultured into 3D cell models, and although many cell lines can form dense spheroids with the help of ECM substrates or scaffolds, cell lines such as SK-BR-3 and suspension cell lines remain difficult to use in the formation of spheroids (Froehlich et al., 2016). In addition, the culture and analysis protocols for 3D cell models have not been standardized; although 3D cell models can be established in large quantities, their formation method, initial cell number, the type and amount of cell-matrix used, and many other factors affect the formation process and lead to the uneven size of 3D cells (Xu et al., 2014; Wan et al., 2017).

## Challenges and opportunities

### The dilemma of tumor organoids

First, due to uncertainty in the growth factors required for some tumor tissues, it is difficult for the corresponding organoids to grow *in vitro* for a long time (Turco et al., 2017; Peng et al., 2018; Zhang et al., 2022b; Geng et al., 2022). Second, at present, tumor organoids are mainly derived from epithelial tumors, and methods for generating nonepithelial cell-derived organoids still need further research (Lombaert et al., 2017; Nikolić and Rawlins, 2017; Martignani et al., 2018). In addition, during organoid culture, growth factors or small molecule inhibitors need to be added to the culture medium, and the requirements for the culture medium are different due to the differences in gene expression in different tumor subtypes, which may affect the gene expression or signal transduction pathway of organoids, which in turn affects drug sensitivity and interferes with the results of the study.

Although organoid technology still has limitations, it provides a new model for tumor research and has great potential. To date, efficient organoid establishment has been achieved in a variety of tumors. Tumor organoids can be used to study the dynamic evolution of tumors and to perform preclinical efficacy evaluation, tumor microenvironment studies, and assessments of adjuvant immunotherapy. With the further development of organoid biobanks and chips, the future use of tumor organoid research is worth investigating during the development of preclinical experiments (Tatullo et al., 2020; Benitez et al., 2021; Zhou et al., 2021).

### The first biobank of tumor organoids was established

Intestinal tumor organoids were the first to be established. Subsequently, the colorectal cancer tumor organoid biobank was established for the first time. Since then, the generation of biobanks of different tumor organoids has begun to develop, and the number has been expanding continuously (Pauli et al., 2017). The Tumor Organoid Biobank contains resources regarding tumor organoids with different pathological types and gene mutations, and tumor organoid studies conducted with large sample sizes can be used to further statistically clarify the relationship between specific gene mutations and drug sensitivity (Pauli et al., 2017; Yang et al., 2021; Ren et al., 2022). The Human Cancer Model Initiative is generating an organoid biobank that provides clinical and genetic information on existing organoids on its website.

### Application in preclinical drug evaluation

Preclinical drug trials focus on clarifying an understanding of drug efficacy and toxicity. Table 2 lists the represented tumor models and their effects. At present, the study of drug efficacy is mostly carried out in animal tumor models. Due to the lack of tumor heterogeneity and the existence of species differences, most drugs show different drug sensitivities *in vivo* and *in vitro* and even in different *in vitro* models (Weeber et al., 2017; Bleijs et al., 2019; Driehuis et al., 2020; Yoshida, 2020). Tumor organoids have high accuracy in predicting a patient’s response to treatment. In colorectal cancer, the patient’s organoid and patient response to the drug were compared, and the results showed that the positive predictive value of the tumor organoid’s response to the patient’s drug was DDA. The study suggests that organoids have shorter incubation times and higher predictive value than traditional models, which helps shorten the drug development cycle. Microfluidic platforms can be used to simulate capillary drug transmission in the tumor microenvironment, providing data regarding drug metabolism and response in cancer patients under physiological flow conditions (Ng et al., 2015). In recent years, a variety of tumor organoids have shown great potential in clinical drug screening, and tumor organoids exhibit drug responses that are consistent with the patient’s drug response and can be used to predict the patient’s treatment response to mitigate the shortcomings of traditional preclinical models in clarifying drug efficacy. It is worth noting that the combination of microfluidic technology and organoid technology can be used to carry out high-throughput drug screening, which greatly shortens the drug development cycle.

**TABLE 2:** Preclinical drug evaluation development in 3D tumor model.

Year	Method	Tumor type	Effect	Reference
2022	Decellularizing and delipidating a porcine breast tissue (TDM) compatible with hydrogel formation	Breast cancer	More closely recreate the breast tumor by incorporating collagen type I (Col1)	Blanco-Fernandez et al. (2022)
2018	Patient-derived pancreatic cancer cells and cancer-associated fibroblasts	Pancreatic cancer	Increase model pathophysiologic relevance, yielding fibroblast-mediated tumor invasion and matrix alignment.	Puls et al. (2018)
2021	Custom 3D printed masks along with simple chemistry modifications to localize hydrophilic “virtual microwells”	Breast cancer cell lines	Tumor response to cisplatin drug treatment, and allows for 3D tumor arrays to be cryopreserved and thawed for on-demand use	Samara et al. (2021)
2019	PANC-1 cells were cultured as tumor spheroids (TSs) using our previously developed mini pillar chips and co-cultured with PSCs, both embedded in collagen gels	Pancreatic ductal adenocarcinoma	Established 3D co-culture of TSs of PANC-1 cells and PSCs using mini pillar histochips as a novel tumoroid model of PDAC	Hwang et al. (2019)
2021	Deconvolute bulk data from endometrial cancers and endometriotic lesions, illuminating the cell types dominating in each of these disorders	Colorectal cancer	Benchmark of the endometrial organoids	Garcia-Alonso et al. (2021)
2018	A living biobank of PDOs from metastatic, gastroesophageal cancer patients in phase I/II clinical trials.	Metastatic gastrointestinal cancers	PDOs could complement existing approaches in defining cancer vulnerabilities and improving treatment responses.	Vlachogiannis et al. (2018)

Abbreviation: PDO, patient-derived organoids; TSs, tumor spheroids; PSCs, pancreatic stellate cells; PDAC, pancreatic ductal adenocarcinoma

### Application in immunotherapy

The development of immunotherapy is gradually changing the treatment strategy that is used in cancer patients. Studies (Jacob et al., 2020; Yuki et al., 2020; Forsythe et al., 2021; Qu et al., 2021) have found that neoantigens associated with tumor cells are key to stimulating an immune response. Insufficient tumor-associated antigens can weaken the proliferation of antitumor immune cells *in vivo*, and in patients with a low tumor mutation burden, *in vitro* activation to amplify immune cells and infusion is a good treatment strategy. Organoids have a highly similar heterogeneity to tumors *in vivo*, and coculture with peripheral blood lymphocytes can induce and enrich reactive S cells in peripheral blood without antigen agnosticism, which is highly targeted for specific individual tumors (Jacob et al., 2020; Yuki et al., 2020; Forsythe et al., 2021; Qu et al., 2021). In the future, chimeric antigen receptor (CAR)-T cell (CART-T) therapy and other cellular immunotherapies in the 3D tumor organoid model platform will be developed for antitumor drug screening (Xu et al., 2018b; Schnalzger et al., 2019; Klein et al., 2020; Yu and Huang, 2020).

In addition, multicellular tumor spheroid culture has 3D characteristics under quiescent culture conditions, but conventional tumor spheroids cannot be used to reflect vascular perfusion or other dynamic characteristics. Some researchers use microfluidic systems to culture multicellular tumor spheroids, but because of the high cost of this approach, it is not suitable for large-scale production, which hinders its application, and the culture method also needs to be further studied. Therefore, 3D tumor cell culture technology still needs to be developed, and when selecting antitumor drugs, it is necessary to consider the conditions and purposes of the experiments and to reasonably select 3D cell culture methods and support material or matrix according to cell type (Edmondson et al., 2014; Fang and Eglen, 2017; Davoudi et al., 2021; Sun et al., 2021). 3D tumor cells mimicking the 3D microenvironment are receiving increasing attention from researchers. In the future, with the continuous improvement of the functionality and controllability of biological materials, materials more suitable for 3D cell culture will likely be prepared according to different research purposes and tumor cell types, such as CloneSeq - Single-cell clonal 3D culture development (Sun et al., 2021). To address the limitations of current 3D tumor cell culture methods and to more accurately simulate the real microenvironment of tumor cells *in vivo*, scientific researchers should aim at obtaining more accurate scientific conclusions in preclinical research and improve antitumor drug screening.

## Conclusion

This report summarizes the 3D tumor organoid model, including the 3D tumor cell culture model, 3D tumor slice model, and organoid culture model. In addition, it provides evidence of the application of the 3D tumor organoid model in precision oncology and drug screening. The aim of the report is to elucidate the value of 3D tumor models in cancer research and provide a preclinical reference for the precise treatment of cancer patients.

## References

[B1] Albanese A. Lam A. K. Sykes E. A. Rocheleau J. V. Chan W. C. (2013). Tumour-on-a-chip provides an optical window into nanoparticle tissue transport. Nat. Commun. 4, 2718. 10.1038/ncomms3718 24177351 PMC3947376

[B2] Anderson N. M. Simon M. C. (2020). The tumor microenvironment. Curr. Biol. 30 (16), R921–r925. 10.1016/j.cub.2020.06.081 32810447 PMC8194051

[B3] Annabi N. Tamayol A. Uquillas J. A. Akbari M. Bertassoni L. E. Cha C. (2014). 25th anniversary article: Rational design and applications of hydrogels in regenerative medicine. Adv. Mat. 26 (1), 85–124. 10.1002/adma.201303233 PMC3925010 24741694

[B4] Aung A. Kumar V. Theprungsirikul J. Davey S. K. Varghese S. (2020). An engineered tumor-on-a-chip device with breast cancer-immune cell interactions for assessing T-cell recruitment. Cancer Res. 80 (2), 263–275. 10.1158/0008-5472.can-19-0342 31744818 PMC8545579

[B5] Bale S. S. Borenstein J. T. (2018). Microfluidic cell culture platforms to capture hepatic physiology and complex cellular interactions. Drug Metab. Dispos. 46 (11), 1638–1646. 10.1124/dmd.118.083055 30115643

[B6] Bartusik-Aebisher D. Chrzanowski G. Bober Z. Aebisher D. (2021). An analytical study of Trastuzumab-dendrimer-fluorine drug delivery system in breast cancer therapy *in vitro* . Biomed. Pharmacother. 133, 111053. 10.1016/j.biopha.2020.111053 33378959

[B7] Benitez J. A. Finlay D. Castanza A. Parisian A. D. Ma J. Longobardi C. (2021). PTEN deficiency leads to proteasome addiction: A novel vulnerability in glioblastoma. Neuro. Oncol. 23 (7), 1072–1086. 10.1093/neuonc/noab001 33428749 PMC8661409

[B117] Blanco-Fernandez B. Rey-Vinolas S. Bağcı G. Rubi-Sans G. Otero J. Navajas D. (2022). Bioprinting decellularized breast tissue for the development of three-dimensional breast cancer models. ACS Appl. Mater. Interfaces. 14 (26), 29467–29482. 35735173 10.1021/acsami.2c00920 PMC9264314

[B8] Bleijs M. van de Wetering M. Clevers H. Drost J. (2019). Xenograft and organoid model systems in cancer research. Embo J. 38 (15), e101654. 10.15252/embj.2019101654 31282586 PMC6670015

[B9] Borodina T. Gileva A. Akasov R. Trushina D. Burov S. Klyachko N. (2021). Fabrication and evaluation of nanocontainers for lipophilic anticancer drug delivery in 3D *in vitro* model. J. Biomed. Mat. Res. 109 (4), 527–537. 10.1002/jbm.b.34721 32945122

[B10] Bromma K. Alhussan A. Perez M. M. Howard P. Beckham W. Chithrani D. B. (2021). Three-dimensional tumor spheroids as a Tool for reliable investigation of combined gold nanoparticle and docetaxel treatment. Cancers (Basel) 13 (6), 1465. 10.3390/cancers13061465 33806801 PMC8004664

[B11] Campisi G. Giannola L. I. Fucarino A. Marino Gammazza A. Pitruzzella A. Marciano V. (2012). Medium-term culture of primary oral squamous cell carcinoma in a three- dimensional model: Effects on cell survival following topical 5-fluororacile delivery by drug-loaded matrix tablets. Curr. Pharm. Des. 18 (34), 5411–5420. 10.2174/138161212803307536 22632387

[B12] Carey-Ewend A. G. Hagler S. B. Bomba H. N. Goetz M. J. Bagó J. R. Hingtgen S. D. (2020) Developing bioinspired three-dimensional models of brain cancer to evaluate tumor-homing neural stem cell therapy. Tissue Eng Part A. Epub ahead of print 2020/10/22. 10.1089/ten.tea.2020.0113 33085922

[B13] Cattaneo C. M. Dijkstra K. K. Fanchi L. F. Kelderman S. Kaing S. van Rooij N. (2020). Tumor organoid-T-cell coculture systems. Nat. Protoc. 15 (1), 15–39. 10.1038/s41596-019-0232-9 31853056 PMC7610702

[B14] Cattin S. Ramont L. Rüegg C. (2018). Characterization and *in vivo* validation of a three-dimensional multi-cellular culture model to study heterotypic interactions in colorectal cancer cell growth, invasion and metastasis. Front. Bioeng. Biotechnol. 6, 97. 10.3389/fbioe.2018.00097 30065926 PMC6056662

[B15] Colombo E. Cattaneo M. G. (2021). Multicellular 3D models to study tumour-stroma interactions. Int. J. Mol. Sci. 22 (4), 1633. 10.3390/ijms22041633 33562840 PMC7915117

[B16] Colosi C. Shin S. R. Manoharan V. Massa S. Costantini M. Barbetta A. (2016). Microfluidic bioprinting of heterogeneous 3D tissue constructs using low-viscosity bioink. Adv. Mat. 28 (4), 677–684. 10.1002/adma.201503310 PMC4804470 26606883

[B17] Davoudi F. Ghorbanpoor S. Yoda S. Pan X. Crowther G. S. Yin X. (2021). Alginate-based 3D cancer cell culture for therapeutic response modeling. Star. Protoc. 2 (2), 100391. 10.1016/j.xpro.2021.100391 33778784 PMC7985559

[B18] De León S. E. Pupovac A. McArthur S. L. (2020). Three-Dimensional (3D) cell culture monitoring: Opportunities and challenges for impedance spectroscopy. Biotechnol. Bioeng. 117 (4), 1230–1240. 10.1002/bit.27270 31956986

[B19] Driehuis E. Kretzschmar K. Clevers H. (2020). Establishment of patient-derived cancer organoids for drug-screening applications. Nat. Protoc. 15 (10), 3380–3409. 10.1038/s41596-020-0379-4 32929210

[B20] Du Y. Herath S. C. B. Wang Q. G. Asada H. Chen P. (2018). Determination of Green's function for three-dimensional traction force reconstruction based on geometry and boundary conditions of cell culture matrices. Acta Biomater. 67, 215–228. 10.1016/j.actbio.2017.12.002 29242157

[B21] Dwyer A. R. Ellies L. G. Holme A. L. Pixley F. J. (2016). A three-dimensional co-culture system to investigate macrophage-dependent tumor cell invasion. J. Biol. Methods 3 (3), e49. 10.14440/jbm.2016.132 31453214 PMC6706153

[B22] Edmondson R. Broglie J. J. Adcock A. F. Yang L. (2014). Three-dimensional cell culture systems and their applications in drug discovery and cell-based biosensors. Assay. Drug Dev. Technol. 12 (4), 207–218. 10.1089/adt.2014.573 24831787 PMC4026212

[B23] Fang Y. Eglen R. M. (2017). Three-dimensional cell cultures in drug discovery and development. SLAS Discov. 22 (5), 456–472. 10.1177/1087057117696795 28520521 PMC5448717

[B24] Flint L. E. Hamm G. Ready J. D. Ling S. Duckett C. J. Cross N. A. (2020). Characterization of an aggregated three-dimensional cell culture model by multimodal mass spectrometry imaging. Anal. Chem. 92 (18), 12538–12547. 10.1021/acs.analchem.0c02389 32786495 PMC7497704

[B25] Foglietta F. Serpe L. Canaparo R. (2021). The effective combination between 3D cancer models and stimuli-responsive nanoscale drug delivery systems. Cells 10 (12), 3295. 10.3390/cells10123295 34943803 PMC8699241

[B26] Forsythe S. D. Erali R. A. Sasikumar S. Laney P. Shelkey E. D'Agostino R. (2021). Organoid platform in preclinical investigation of personalized immunotherapy efficacy in appendiceal cancer: Feasibility study. Clin. Cancer Res. 27 (18), 5141–5150. 10.1158/1078-0432.ccr-21-0982 34210684 PMC8720262

[B27] Froehlich K. Haeger J. D. Heger J. Pastuschek J. Photini S. M. Yan Y. (2016). Generation of multicellular breast cancer tumor spheroids: Comparison of different protocols. J. Mammary Gland. Biol. Neoplasia 21 (3-4), 89–98. 10.1007/s10911-016-9359-2 27518775

[B28] Garcia-Alonso L. Handfield L. F. Roberts K. Nikolakopoulou K. Fernando R. C. Gardner L. (2021). Mapping the temporal and spatial dynamics of the human endometrium *in vivo* and *in vitro* . Nat. Genet. 53 (12), 1698–1711. 10.1038/s41588-021-00972-2 34857954 PMC8648563

[B29] Geng R. Harland N. Montes-Mojarro I. A. Fend F. Aicher W. K. Stenzl A. (2022). CD24: A marker for an extended expansion potential of urothelial cancer cell organoids *in vitro*? Int. J. Mol. Sci. 23 (10), 5453. 10.3390/ijms23105453 35628262 PMC9141653

[B30] Godugu C. Patel A. R. Desai U. Andey T. Sams A. Singh M. (2013). AlgiMatrix™ based 3D cell culture system as an *in-vitro* tumor model for anticancer studies. PLoS One 8 (1), e53708. 10.1371/journal.pone.0053708 23349734 PMC3548811

[B31] Godugu C. Singh M. (2016). AlgiMatrix™-Based 3D cell culture system as an *in vitro* tumor model: An important Tool in cancer research. Methods Mol. Biol. 1379, 117–128. 10.1007/978-1-4939-3191-0_11 26608295

[B32] Gungor-Ozkerim P. S. Inci I. Zhang Y. S. Khademhosseini A. Dokmeci M. R. (2018). Bioinks for 3D bioprinting: An overview. Biomater. Sci. 6 (5), 915–946. 10.1039/c7bm00765e 29492503 PMC6439477

[B33] Habanjar O. Diab-Assaf M. Caldefie-Chezet F. Delort L. (2021). 3D cell culture systems: Tumor application, advantages, and disadvantages. Int. J. Mol. Sci. 22 (22), 12200. 10.3390/ijms222212200 34830082 PMC8618305

[B34] Heinrich M. A. Liu W. Jimenez A. Yang J. Akpek A. Liu X. (2019). Bioprinting: 3D bioprinting: From benches to translational applications (small 23/2019). Small 15 (23), 1970126. 10.1002/smll.201970126 PMC6752725 31033203

[B35] Horie M. Saito A. Mikami Y. Ohshima M. Morishita Y. Nakajima J. (2012). Characterization of human lung cancer-associated fibroblasts in three-dimensional *in vitro* co-culture model. Biochem. Biophys. Res. Commun. 423 (1), 158–163. 10.1016/j.bbrc.2012.05.104 22634307

[B36] Horie M. Saito A. Yamaguchi Y. Ohshima M. Nagase T. (2015). Three-dimensional Co-culture model for tumor-stromal interaction. J. Vis. Exp. (96), 52469. Epub ahead of print 2015/03/06. 10.3791/52469 PMC4354609 25741617

[B37] Hsu S. H. Huang G. S. (2013). Substrate-dependent Wnt signaling in MSC differentiation within biomaterial-derived 3D spheroids. Biomaterials 34 (20), 4725–4738. 10.1016/j.biomaterials.2013.03.031 23562051

[B38] Huang S. Zhao Q. (2020). Nanomedicine-combined immunotherapy for cancer. Curr. Med. Chem. 27 (34), 5716–5729. 10.2174/0929867326666190618161610 31250752

[B39] Huang S. Zhao Q. (2022). The trend of immunotherapy combined with nanomedicine. Curr. Med. Chem. 29 (22), 3817–3818. 10.2174/0929867328666211117094947 34789123

[B40] Huh D. Hamilton G. A. Ingber D. E. (2011). From 3D cell culture to organs-on-chips. Trends Cell Biol. 21 (12), 745–754. 10.1016/j.tcb.2011.09.005 22033488 PMC4386065

[B118] Hwang H. J. Oh M. S. Lee D. W. Kuh H.-J. (2019). Multiplex quantitative analysis of stroma-mediated cancer cell invasion, matrix remodeling, and drug response in a 3D co-culture model of pancreatic tumor spheroids and stellate cells. J. Exp. Clin. Cancer Res. 38 (1), 258. 31200779 10.1186/s13046-019-1225-9 PMC6567511

[B41] Ilina O. Gritsenko P. G. Syga S. Lippoldt J. La Porta C. A. M. Chepizhko O. (2020). Cell-cell adhesion and 3D matrix confinement determine jamming transitions in breast cancer invasion. Nat. Cell Biol. 22 (9), 1103–1115. 10.1038/s41556-020-0552-6 32839548 PMC7502685

[B42] Ingeson-Carlsson C. Martinez-Monleon A. Nilsson M. (2015). Differential effects of MAPK pathway inhibitors on migration and invasiveness of BRAF(V600E) mutant thyroid cancer cells in 2D and 3D culture. Exp. Cell Res. 338 (2), 127–135. 10.1016/j.yexcr.2015.08.003 26384551

[B43] Jacob F. Ming G. L. Song H. (2020). Generation and biobanking of patient-derived glioblastoma organoids and their application in CAR T cell testing. Nat. Protoc. 15 (12), 4000–4033. 10.1038/s41596-020-0402-9 33169003

[B44] Jensen C. Teng Y. (2020). Is it time to start transitioning from 2D to 3D cell culture? Front. Mol. Biosci. 7, 33. 10.3389/fmolb.2020.00033 32211418 PMC7067892

[B45] Kang J. Lee D. W. Hwang H. J. Yeon S. E. Lee M. Y. Kuh H. J. (2016). Mini-pillar array for hydrogel-supported 3D culture and high-content histologic analysis of human tumor spheroids. Lab. Chip 16 (12), 2265–2276. 10.1039/c6lc00526h 27194205

[B46] Kenerson H. L. Sullivan K. M. Labadie K. P. Pillarisetty V. G. Yeung R. S. (2021). Protocol for tissue slice cultures from human solid tumors to study therapeutic response. Star. Protoc. 2 (2), 100574. 10.1016/j.xpro.2021.100574 34142099 PMC8184656

[B47] Kim J. Y. Kim H. W. Bae S. J. Joo D. Huh K. Fang Y. (2012). Hybrid cellular spheroids from hepatocellular carcinoma and insulin-secreting cell lines. Transpl. Proc. 44 (4), 1095–1098. 10.1016/j.transproceed.2012.02.016 22564634

[B48] Klein E. Hau A. C. Oudin A. Golebiewska A. Niclou S. P. (2020). Glioblastoma organoids: Pre-clinical applications and challenges in the context of immunotherapy. Front. Oncol. 10, 604121. 10.3389/fonc.2020.604121 33364198 PMC7753120

[B49] Lee G. Y. Kenny P. A. Lee E. H. Bissell M. J. (2007). Three-dimensional culture models of normal and malignant breast epithelial cells. Nat. Methods 4 (4), 359–365. 10.1038/nmeth1015 17396127 PMC2933182

[B50] Li J. Zhou Y. Chen W. Yuan Z. You B. Liu Y. (2018). A novel 3D *in vitro* tumor model based on silk fibroin/chitosan scaffolds to mimic the tumor microenvironment. ACS Appl. Mat. Interfaces 10 (43), 36641–36651. 10.1021/acsami.8b10679 30360129

[B51] Lombaert I. Movahednia M. M. Adine C. Ferreira J. N. (2017). Concise review: Salivary Gland regeneration: Therapeutic approaches from stem cells to tissue organoids. Stem Cells 35 (1), 97–105. 10.1002/stem.2455 27406006 PMC6310135

[B52] Lu Z. Nie B. Zhai W. Hu Z. (2021). Delineating the longitudinal tumor evolution using organoid models. J. Genet. Genomics 48 (7), 560–570. 10.1016/j.jgg.2021.06.010 34366272

[B53] Lugo-Cintrón K. M. Gong M. M. Ayuso J. M. Tomko L. A. Beebe D. J. Virumbrales-Munoz M. (2020). Breast fibroblasts and ECM components modulate breast cancer cell migration through the secretion of MMPs in a 3D microfluidic Co-culture model. Cancers (Basel) 12 (5), 1173. 10.3390/cancers12051173 PMC7281408 32384738

[B54] Mandrycky C. Wang Z. Kim K. Kim D. H. (2016). 3D bioprinting for engineering complex tissues. Biotechnol. Adv. 34 (4), 422–434. 10.1016/j.biotechadv.2015.12.011 26724184 PMC4879088

[B55] Martignani E. Accornero P. Miretti S. Baratta M. (2018). Bovine mammary organoids: A model to study epithelial mammary cells. Methods Mol. Biol. 1817, 137–144. 10.1007/978-1-4939-8600-2_14 29959710

[B56] Mehling M. Tay S. (2014). Microfluidic cell culture. Curr. Opin. Biotechnol. 25, 95–102. 10.1016/j.copbio.2013.10.005 24484886

[B57] Murphy S. V. De Coppi P. Atala A. (2020). Opportunities and challenges of translational 3D bioprinting. Nat. Biomed. Eng. 4 (4), 370–380. 10.1038/s41551-019-0471-7 31695178

[B58] Nath S. Devi G. R. (2016). Three-dimensional culture systems in cancer research: Focus on tumor spheroid model. Pharmacol. Ther. 163, 94–108. 10.1016/j.pharmthera.2016.03.013 27063403 PMC4961208

[B59] Neal J. T. Li X. Zhu J. Giangarra V. Grzeskowiak C. L. Ju J. (2018). Organoid modeling of the tumor immune microenvironment. Cell 175 (7), 1972–1988.e16. 10.1016/j.cell.2018.11.021.1972 30550791 PMC6656687

[B60] Ng A. H. Li B. B. Chamberlain M. D. Wheeler A. R. (2015). Digital microfluidic cell culture. Annu. Rev. Biomed. Eng. 17, 91–112. 10.1146/annurev-bioeng-071114-040808 26643019

[B61] Nii T. Kuwahara T. Makino K. Tabata Y. (2020). A Co-culture system of three-dimensional tumor-associated macrophages and three-dimensional cancer-associated fibroblasts combined with biomolecule release for cancer cell migration. Tissue Eng. Part A 26 (23-24), 1272–1282. 10.1089/ten.tea.2020.0095 32434426

[B62] Nikolić M. Z. Rawlins E. L. (2017). Lung organoids and their use to study cell-cell interaction. Curr. Pathobiol. Rep. 5 (2), 223–231. 10.1007/s40139-017-0137-7 28596933 PMC5446548

[B63] Nishida-Aoki N. Bondesson A. J. Gujral T. S. (2020). Measuring real-time drug response in organotypic tumor tissue slices. J. Vis. Exp. (159), e61036. Epub ahead of print 2020/05/19. 10.3791/61036 32420994

[B64] Nuciforo S. Fofana I. Matter M. S. Blumer T. Calabrese D. Boldanova T. (2018). Organoid models of human liver cancers derived from tumor needle biopsies. Cell Rep. 24 (5), 1363–1376. 10.1016/j.celrep.2018.07.001 30067989 PMC6088153

[B65] Pang L. Ding J. Ge Y. Fan J. Fan S. K. (2019). Single-cell-derived tumor-sphere formation and drug-resistance assay using an integrated microfluidics. Anal. Chem. 91 (13), 8318–8325. 10.1021/acs.analchem.9b01084 31148455

[B66] Pauli C. Hopkins B. D. Prandi D. Shaw R. Fedrizzi T. Sboner A. (2017). Personalized *in vitro* and *in vivo* cancer models to guide precision medicine. Cancer Discov. 7 (5), 462–477. 10.1158/2159-8290.cd-16-1154 28331002 PMC5413423

[B67] Peng W. C. Logan C. Y. Fish M. Anbarchian T. Aguisanda F. Alvarez-Varela A. (2018). Inflammatory cytokine TNFα promotes the long-term expansion of primary hepatocytes in 3D culture. Cell 175 (6), 1607–1619.e15. e1615. 10.1016/j.cell.2018.11.012 30500539 PMC6497386

[B68] Peng Z. Lv X. Huang S. (2022). Recent progress on the role of fibronectin in tumor stromal immunity and immunotherapy. Curr. Top. Med. Chem. 22, 647. Epub ahead of print 2022/06/17. 10.2174/1568026622666220615152647 35708087

[B69] Polidoro M. A. Ferrari E. Marzorati S. Lleo A. Rasponi M. (2021). Experimental liver models: From cell culture techniques to microfluidic organs-on-chip. Liver Int. 41 (8), 1744–1761. 10.1111/liv.14942 33966344

[B119] Puls T. J. Tan X. Husain M. Whittington C. F. Fishel M. Voytik-Harbin S. L. (2018). Development of a novel 3D tumor-tissue invasion model for high-throughput, High-content phenotypic drug screening. Sci. Rep. 8 (1), 13039. 30158688 10.1038/s41598-018-31138-6 PMC6115445

[B70] Qu J. Kalyani F. S. Liu L. Cheng T. Chen L. (2021). Tumor organoids: Synergistic applications, current challenges, and future prospects in cancer therapy. Cancer Commun. (Lond). 41 (12), 1331–1353. 10.1002/cac2.12224 34713636 PMC8696219

[B71] Rashidian J. Luo K. (2016). Three-dimensional mammary epithelial cell morphogenesis model for analysis of TGFß signaling. Methods Mol. Biol. 1344, 121–135. 10.1007/978-1-4939-2966-5_7 26520121

[B72] Ravi M. Paramesh V. Kaviya S. R. Anuradha E. Solomon F. P. (2015). 3D cell culture systems: Advantages and applications. J. Cell. Physiol. 230 (1), 16–26. 10.1002/jcp.24683 24912145

[B73] Ren X. Chen W. Yang Q. Li X. Xu L. (2022). Patient-derived cancer organoids for drug screening: Basic technology and clinical application. J. Gastroenterol. Hepatol. 37, 1446–1454. Epub ahead of print 2022/07/01. 10.1111/jgh.15930 35771719

[B74] Rodenhizer D. Gaude E. Cojocari D. Mahadevan R. Frezza C. Wouters B. G. (2016). A three-dimensional engineered tumour for spatial snapshot analysis of cell metabolism and phenotype in hypoxic gradients. Nat. Mat. 15 (2), 227–234. 10.1038/nmat4482 PMC5214740 26595121

[B75] Rodenhizer D. Dean T. Xu B. Cojocari D. McGuigan A. P. (2018). A three-dimensional engineered heterogeneous tumor model for assessing cellular environment and response. Nat. Protoc. 13 (9), 1917–1957. 10.1038/s41596-018-0022-9 30190554

[B76] Rodríguez-Dorantes M. Cruz-Hernandez C. D. Cortés-Ramírez S. A. Cruz-Burgos J. M. Reyes-Grajeda J. P. Peralta-Zaragoza O. (2021). Prostate cancer spheroids: A three-dimensional model for studying tumor heterogeneity. Methods Mol. Biol. 2174, 13–17. 10.1007/978-1-0716-0759-6_2 32813241

[B77] Salgueiredo-Giudice F. Corrêa-Abrahão A. Fornias-Sperandio F. da-Costa-Dal-Vechio A. dos-Santos-Pinto-Junior D. (2012). An *in vitro* study showing the three-dimensional microenvironment influence over the behavior of head and neck squamous cell carcinoma. Med. Oral Patol. Oral Cir. Bucal 17 (3), e377–e382. 10.4317/medoral.17538 22143720 PMC3476095

[B120] Samara B. Deliorman M. Sukumar P. Qasaimeh M. A. (2021). Cryopreservable arrays of paper-based 3D tumor models for high throughput drug screening. Lab. Chip. 21 (5), 844–854. 33615319 10.1039/d0lc01300e

[B78] Schnalzger T. E. de Groot M. H. Zhang C. Mosa M. H. Michels B. E. Roder J. (2019). 3D model for CAR-mediated cytotoxicity using patient-derived colorectal cancer organoids. Embo J. 38 (12), e100928. 10.15252/embj.2018100928 31036555 PMC6576164

[B79] Schreiber-Brynzak E. Klapproth E. Unger C. Lichtscheidl-Schultz I. Goschl S. Schweighofer S. (2015). Three-dimensional and co-culture models for preclinical evaluation of metal-based anticancer drugs. Invest. New Drugs 33 (4), 835–847. 10.1007/s10637-015-0260-4 26091914

[B80] Sharonov G. V. Serebrovskaya E. O. Yuzhakova D. V. Britanova O. V. Chudakov D. M. (2020). B cells, plasma cells and antibody repertoires in the tumour microenvironment. Nat. Rev. Immunol. 20 (5), 294–307. 10.1038/s41577-019-0257-x 31988391

[B81] Shin C. S. Kwak B. Han B. Park K. (2013). Development of an *in vitro* 3D tumor model to study therapeutic efficiency of an anticancer drug. Mol. Pharm. 10 (6), 2167–2175. 10.1021/mp300595a 23461341 PMC3880422

[B82] Sivakumar R. Chan M. Shin J. S. Nishida-Aoki N. Kenerson H. L. Elemento O. (2019). Organotypic tumor slice cultures provide a versatile platform for immuno-oncology and drug discovery. Oncoimmunology 8 (12), e1670019. 10.1080/2162402x.2019.1670019 31741771 PMC6844320

[B83] Sobierajska K. Ciszewski W. M. Sacewicz-Hofman I. Niewiarowska J. (2020). “Endothelial cells in the tumor microenvironment,” in Tumor microenvironment: Non-hematopoietic cells. Editor Birbrair A. (Cham: Springer International Publishing), 71–86. 10.1007/978-3-030-37184-5_6 32040856

[B84] Sun X. Bavli D. Kozulin C. Motzik A. Buxboim A. Ram O. (2021). CloneSeq - single-cell clonal 3D culture and analysis protocol. Star. Protoc. 2 (4), 100794. 10.1016/j.xpro.2021.100794 34632413 PMC8488600

[B85] Swann J. B. Smyth M. J. (2007). Immune surveillance of tumors. J. Clin. Invest. 117 (5), 1137–1146. 10.1172/jci31405 17476343 PMC1857231

[B86] Tatullo M. Marrelli B. Benincasa C. Aiello E. Makeeva I. Zavan B. (2020). Organoids in translational oncology. J. Clin. Med. 9 (9), 2774. 10.3390/jcm9092774 PMC7564148 32867142

[B87] Tortorella E. Palmieri D. Piermarini M. Gigante D. Oddo L. Toumia Y. (2021). Phase change dimethyldioctadecylammonium-shelled microdroplets as a promising drug delivery system: Results on 3D spheroids of mammalian tumor cells. J. Vis. Exp. (169), e62255. Epub ahead of print 2021/03/30. 10.3791/62255 33779605

[B88] Truffi M. Sorrentino L. Corsi F. (2020). “Fibroblasts in the tumor microenvironment,” in Tumor microenvironment: Non-hematopoietic cells. Editor Birbrair A. (Cham: Springer International Publishing), 15–29. 10.1007/978-3-030-37184-5_2 32040852

[B89] Tsai K. K. Huang S. S. Northey J. J. Liao W. Y. Hsu C. C. Cheng L. H. (2022). Screening of organoids derived from patients with breast cancer implicates the repressor NCOR2 in cytotoxic stress response and antitumor immunity. Nat. Cancer 3 (6), 734–752. 10.1038/s43018-022-00375-0 35618935 PMC9246917

[B90] Tung Y-C. Hsiao A. Y. Allen S. G. Torisawa Y. s. Ho M. Takayama S. (2011). High-throughput 3D spheroid culture and drug testing using a 384 hanging drop array. Analyst 136 (3), 473–478. 10.1039/c0an00609b 20967331 PMC7454010

[B91] Turco M. Y. Gardner L. Hughes J. Cindrova-Davies T. Gomez M. J. Farrell L. (2017). Long-term, hormone-responsive organoid cultures of human endometrium in a chemically defined medium. Nat. Cell Biol. 19 (5), 568–577. 10.1038/ncb3516 28394884 PMC5410172

[B92] Turley S. J. Cremasco V. Astarita J. L. (2015). Immunological hallmarks of stromal cells in the tumour microenvironment. Nat. Rev. Immunol. 15 (11), 669–682. 10.1038/nri3902 26471778

[B93] Tuveson D. Clevers H. (2019). Cancer modeling meets human organoid technology. Science 364 (6444), 952–955. 10.1126/science.aaw6985 31171691

[B94] van de Wetering M. Francies H. E. Francis J. M. Bounova G. Iorio F. Pronk A. (2015). Prospective derivation of a living organoid biobank of colorectal cancer patients. Cell 161 (4), 933–945. 10.1016/j.cell.2015.03.053 25957691 PMC6428276

[B95] Velez D. O. Tsui B. Goshia T. Chute C. L. Han A. Carter H. (2017). 3D collagen architecture induces a conserved migratory and transcriptional response linked to vasculogenic mimicry. Nat. Commun. 8 (1), 1651. 10.1038/s41467-017-01556-7 29162797 PMC5698427

[B96] Vinci M. Box C. Eccles S. A. (2015). Three-dimensional (3D) tumor spheroid invasion assay. J. Vis. Exp. (99), e52686. Epub ahead of print 2015/05/21. 10.3791/52686 25993495 PMC4542056

[B121] Vlachogiannis G. Hedayat S. Vatsiou A. Jamin Y. Fernández-Mateos J. Khan K. (2018). Patient-derived organoids model treatment response of metastatic gastrointestinal cancers. Sci. 359(6378), 920–926. 10.1126/science.aao2774 PMC6112415 29472484

[B97] Walsh C. L. Babin B. M. Kasinskas R. W. Foster J. A. McGarry M. J. Forbes N. S. (2009). A multipurpose microfluidic device designed to mimic microenvironment gradients and develop targeted cancer therapeutics. Lab. Chip 9 (4), 545–554. 10.1039/b810571e 19190790 PMC2855303

[B98] Wan X. Ball S. Willenbrock F. Yeh S. Vlahov N. Koennig D. (2017). Perfused three-dimensional organotypic culture of human cancer cells for therapeutic evaluation. Sci. Rep. 7 (1), 9408. 10.1038/s41598-017-09686-0 28842598 PMC5573358

[B99] Wang K. Zhang X. Liu Y. Liu C. Jiang B. Jiang Y. (2014). Tumor penetrability and anti-angiogenesis using iRGD-mediated delivery of doxorubicin-polymer conjugates. Biomaterials 35 (30), 8735–8747. 10.1016/j.biomaterials.2014.06.042 25023394

[B100] Weeber F. Ooft S. N. Dijkstra K. K. Voest E. E. (2017). Tumor organoids as a pre-clinical cancer model for drug discovery. Cell Chem. Biol. 24 (9), 1092–1100. 10.1016/j.chembiol.2017.06.012 28757181

[B101] Xing F. Liu Y. C. Huang S. Lyu X. Su S. M. Chan U. I. (2021). Accelerating precision anti-cancer therapy by time-lapse and label-free 3D tumor slice culture platform. Theranostics 11 (19), 9415–9430. 10.7150/thno.59533 34646378 PMC8490519

[B102] Xu X. Farach-Carson M. C. Jia X. (2014). Three-dimensional *in vitro* tumor models for cancer research and drug evaluation. Biotechnol. Adv. 32 (7), 1256–1268. 10.1016/j.biotechadv.2014.07.009 25116894 PMC4171250

[B103] Xu H. Jiao Y. Qin S. Zhao W. Chu Q. Wu K. (2018a). Organoid technology in disease modelling, drug development, personalized treatment and regeneration medicine. Exp. Hematol. Oncol. 7, 30. 10.1186/s40164-018-0122-9 30534474 PMC6282260

[B104] Xu H. Lyu X. Yi M. Zhao W. Song Y. Wu K. (2018b). Organoid technology and applications in cancer research. J. Hematol. Oncol. 11 (1), 116. 10.1186/s13045-018-0662-9 30219074 PMC6139148

[B105] Xu R. Zhou X. Wang S. Trinkle C. (2021). Tumor organoid models in precision medicine and investigating cancer-stromal interactions. Pharmacol. Ther. 218, 107668. 10.1016/j.pharmthera.2020.107668 32853629 PMC7855432

[B106] Yan H. H. N. Siu H. C. Law S. Ho S. L. Yue S. S. Tsui W. Y. (2018). A comprehensive human gastric cancer organoid biobank captures tumor subtype heterogeneity and enables therapeutic screening. Cell Stem Cell 23 (6), 882–897.e11. e811. 10.1016/j.stem.2018.09.016 30344100

[B107] Yang H. Wang Y. Wang P. Zhang N. (2021). Tumor organoids for cancer research and personalized medicine. Cancer Biol. Med. 19 (3), 0–332. 10.20892/j.issn.2095-3941.2021.0335 PMC8958892 34520134

[B108] Yi C. Lai S. L. Tsang C. M. Artemenko M. Shuen Tang M. K. Pang S. W. (2021). A three-dimensional spheroid-specific role for Wnt-β-catenin and Eph-ephrin signaling in nasopharyngeal carcinoma cells. J. Cell Sci. 134 (16), jcs256461. 10.1242/jcs.256461 34338780

[B109] Yoshida G. J. (2020). Applications of patient-derived tumor xenograft models and tumor organoids. J. Hematol. Oncol. 13 (1), 4. 10.1186/s13045-019-0829-z 31910904 PMC6947974

[B110] Yu J. Huang W. (2020). The progress and clinical application of breast cancer organoids. Int. J. Stem Cells 13 (3), 295–304. 10.15283/ijsc20082 32840232 PMC7691857

[B111] Yuki K. Cheng N. Nakano M. Kuo C. J. (2020). Organoid models of tumor immunology. Trends Immunol. 41 (8), 652–664. 10.1016/j.it.2020.06.010 32654925 PMC7416500

[B112] Zaki M. Y. W. Shetty S. Wilkinson A. L. Patten D. A. Oakley F. Reeves H. (2021). A three-dimensional spheroid model to investigate the tumor-stromal interaction in hepatocellular carcinoma. J. Vis. Exp. (175), e62868. Epub ahead of print 2021/10/19. 10.3791/62868 34661571

[B113] Zhang J. Tavakoli H. Ma L. Li X. Han L. Li X. (2022a). Immunotherapy discovery on tumor organoid-on-a-chip platforms that recapitulate the tumor microenvironment. Adv. Drug Deliv. Rev. 187, 114365. 10.1016/j.addr.2022.114365 35667465

[B114] Zhang M. Lv L. Cai H. Li Y. Gao F. Yu L. (2022b). Long-term expansion of porcine intestinal organoids serves as an *in vitro* model for swine enteric coronavirus infection. Front. Microbiol. 13, 865336. 10.3389/fmicb.2022.865336 35369438 PMC8967161

[B115] Zhou L. Zhang C. Zhang Y. Shi C. (2021). Application of organoid models in prostate cancer research. Front. Oncol. 11, 736431. 10.3389/fonc.2021.736431 34646778 PMC8504437

[B116] Zuppinger C. (2019). 3D cardiac cell culture: A critical review of current technologies and applications. Front. Cardiovasc. Med. 6, 87. 10.3389/fcvm.2019.00087 31294032 PMC6606697

